# Emergency medicine residency training in Africa: overview of curriculum

**DOI:** 10.1186/s12909-019-1729-1

**Published:** 2019-07-31

**Authors:** Hendry R. Sawe, Abena Akomeah, Juma A. Mfinanga, Michael S. Runyon, Erin Noste

**Affiliations:** 1 Emergency Medicine Department, Muhimbili University of Health and Allied Sciences, P.O. Box 65001, Dar es salaam, Tanzania; 2 Emergency Medicine Department, Muhimbili National Hospital, Dar es Salaam, Tanzania; 3 Emergency Department, University of Maryland, Baltimore, MD USA; 4 Emergency Department, Carolinas Medical Center Main, Charlotte, North Carolina USA

**Keywords:** Emergency medicine, Emergency training, Residency, Africa

## Abstract

**Background:**

Emergency Medicine (EM) is a rapidly developing specialty in Africa with several emergency medicine residency-training programs (EMRPs) established across the continent over the past decade. Despite rapid proliferation of the specialty, little is known about emergency care curriculum structure and content. We provide an overview of Africa’s EMRPs.

**Methods:**

This was a descriptive cross-sectional survey conducted of EMRPs in Africa between January 2017 and December 2017. Data were prospectively collected using a structured survey that was developed and administered through online data capture software, REDCap (Version 7.2.2, Vanderbilt, Nashville, TN, USA). Survey questions focused on curriculum structure and design, including clinical rotations, didactics, research, and evaluation. Data are summarized with descriptive statistics.

**Results:**

The survey was sent to the leadership of 15 EMRPs in 12 different African countries and 11 (73%) responded. Five (46%) of the responding programs were started by local non-EM trained faculty, two (18%) were started by international partners, and the remainder by a combination of local non-EM faculty and international partners. Overall, Seven (64%) of the countries offer a 4-year EMRP. In General, 40% of curriculums are influenced the contents developed by African Federation for Emergency Medicine. All programs offer resident led-didactics, with a median of 12 h (Interquartile range 9–6 h) per month. All EMRPs have a mandatory research requirement. All EMRPs offer clinical rotations in the ED, Paediatrics, and Obstetrics and Gynaecology, while only 2 programs offer rotations in radiology and neonatal intensive care units. Only 46% of EMRPs have in-ED clinical supervision by specialist.

**Conclusion:**

The EMRPs in Africa were started by non-EM trained local faculty alone or collaboration with international partners. The curriculum offers most exposure to ED, and less exposure in radiology and neonatal intensive care. Residents are highly involved in leading didactics and less than half of the programs have in-ED specialist supervision of patient care.

**Electronic supplementary material:**

The online version of this article (10.1186/s12909-019-1729-1) contains supplementary material, which is available to authorized users.

## Background

Emergency Medicine (EM) is a rapidly evolving specialty in Africa, despite being a relatively new concept in many countries [1]. Developing high quality emergency care systems (ECS) that can impact patient outcomes is complex, and even more so in low resourced environments [2]. Studies on ECS development show that irrespective of culture and socioeconomic differences most ECS systems will go through different stages of maturation to include academic development, patient care and management systems development, economic and legislative structure building, and national health policy development [3–5]. The EM training throughout Africa is at different stages, there are several countries that provide midlevel training in emergency medicine to general practitioners, nurses, and nurse practitioners or offer paramedic training [6–8], however there is little data on the sustainability and impact of these programs.

Specialty training in EM is an essential component of high-quality emergency care systems in any country. However, several challenges exists in different African countries, ranging from lack of formal pre-hospital systems, inadequate infrastructure to support EM practice and training, and a general lack of sufficient local expertise to train and provide clinical oversight to EM trainees [4].

As of 2017 there were at least twelve African countries with emergency medicine residency programs (EMRPs). These countries started their EMRPs at different times, with South Africa the first to launch a dedicated emergency medicine specialist-training program in 2004 [9]. Since then, several other African countries including Botswana, Egypt, Ethiopia, Ghana, Kenya, Libya, Malawi, Mozambique, Rwanda, Sudan and Tanzania have all developed their programs, and are at different stages of implementation. The formation of the African Federation for Emergency Medicine (AFEM) in 2009 provided an opportunity for professional linkages and exchange of scientific content and expertise among the different training programs across Africa [10].

Despite rapid proliferation of the specialty, little is known about emergency care training curricula in different EMRPs across Africa. We aimed to provide an overview of Africa’s EMRP curriculum structure and content. This will provide essential data on the current state of emergency medicine residency curricula across Africa, paving the way for potential future standardization of training as appropriate.

## Methods

### Study design

This was a descriptive cross-sectional survey conducted of EMRPs in Africa between January 2017 and December 2017. The study was carried out as part of a project focused on reviewing the curricula and organisation of EMRPs in Africa.

#### Study setting and population

This survey was administered to EMRP program leadership (chairs, program directors, assistant program directors) across Africa. During the study period, 15 EMRPs spanning across 12 countries were identified, namely Botswana, Egypt, Ethiopia, Ghana, Kenya, Libya, Malawi, Mozambique, Rwanda, South Africa, Sudan and Tanzania. All countries had one program each except Egypt and South Africa, which had two and three EMRPs respectively. EMRPs were defined as post-graduate medical training programs of at least 18 months duration leading to a degree, diploma, or certificate of recognized specialization in emergency medicine.

#### Data collection and analysis

The survey questions focused on curriculum structure: clinical rotations, didactics, research, evaluation and curriculum design (Additional file 1). Data are summarized as frequencies and percentages, means and medians. One author (AK) conducted a search to identify all African EMRP programs through different online sources (African Journal of Emergency medicine (AFJEM), Google, Medline and EMBASE), and another author (HRS) contacted EM associations across Africa through the African Federation for Emergency Medicine (AFEM) network for information on EMRPs and the contact information for program leadership. After identifying the total number of countries with EMRPs, the leadership of each program (including chair of the department and residency program directors) were sent a message describing the project as well as a link to the survey online. Non-responders were reminded at least three times, on different occasions, over a period of six months. Data collection was conducted using an online survey that was built with the REDCap electronic data capture software (Version 7.2.2, Vanderbilt, Nashville, TN, USA). Procedure, frequency and univariate functions were performed to check for any outliers and clean the dataset. Descriptive statistics, including means, standard deviations, medians, and ranges were calculated.

## Results

**Fig. 1:**
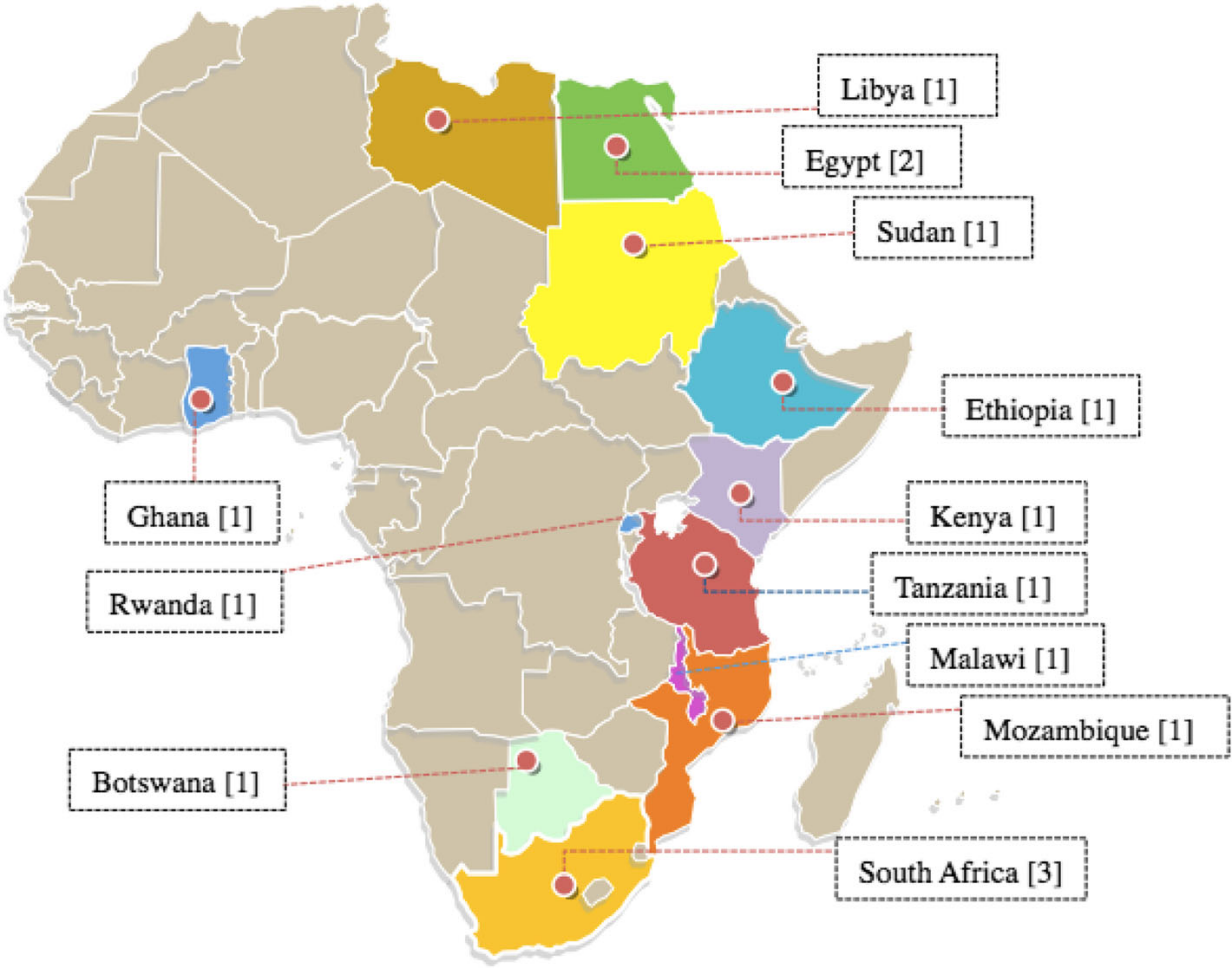
Map of Africa showing countries with EMRPs

During the study period there were 15 African EMRPs for physicians identified across 12 different countries. Survey was sent to all programs and we received 11 responses from 10 different countries, representing response rates of 73% of programs and 83% of countries respectively Fig. 1.

### Curriculum development, program duration and entry requirement

Overall, Five (46%) of the responding programs were started by local non-EM trained faculty, two (18%) were started by international partners, and the remainder by a combination of local non-EM faculty and international partners. Seven (63%) of the programs offer a 4-year curriculum and the minimum entry for all programs was at least doctor of medicine (or equivalent). Three (27%) have not changed their curriculum since the start of the program. (Libya, Botswana, Tanzania all started after 2010 or later) Table 1.

**Table 1:** Curriculum development program duration and entry requirement

Country	EMRP Pioneer	EMRP Duration in years	Curriculum model	Curriculum changes
Botswana	Local non-EM Faculty	4	South African programs	NO
Egypt*	Local non-EM Faculty	3	Local	YES
Egypt*	Local non-EM Faculty	3	Mixed UK and USA	YES
Ethiopia	Local non-EM & International partners	3	Local	YES
Kenya	International partners	4	Local	YES
Libya	Local non-EM & International partners	4	European programs	NO
Malawi	Local non-EM & International partners	4	South African programs	YES
Rwanda	International partners	4	Local	YES
South Africa	Local non-EM Faculty	4	Australian programs	YES
Sudan	Local non-EM Faculty	4	Local	YES
Tanzania	Local non-EM & International partners	3	Mixed USA & South African Programs	NO

* Two different programs in the same country responded to the survey

**Fig. 2:**
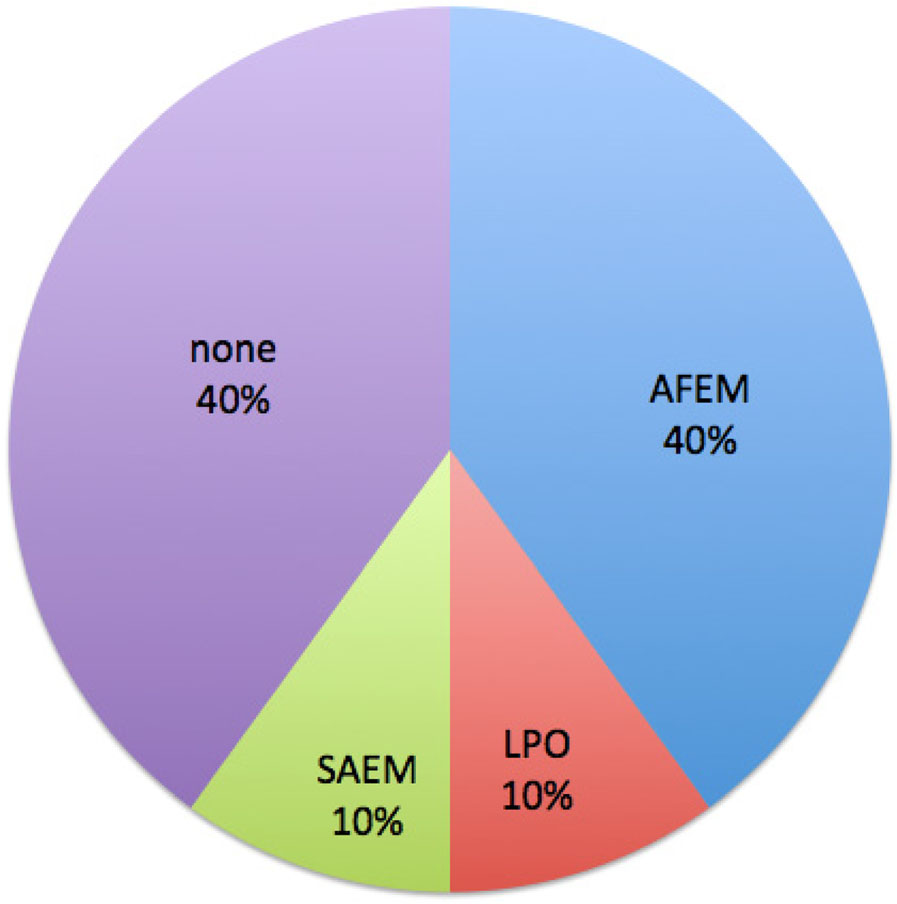
Professional organisations influencing curriculum of EMRPs in Africa

Of the 10 programs providing the details of their curricula, 6 (60%) review their curriculum anywhere from annually to every 5 years, while 4 (40%) have no specific timing for reviewing their curriculum (South Africa, Botswana, Malawi and Tanzania). 60% curricula are also influenced by local and international professional organisations: 4/10 by African Federation for Emergency Medicine (AFEM), 10% Society for Academic Emergency Medicine (SAEM) and 10% by local professional organisation (LPO) Fig. 2.

### Teaching and evaluation

All programs offer didactics, with a median of 12 h (Interquartile range 9–6 h) per month across all EMRPs. All programs allow residents to lecture during these sessions. While all programs mandate research efforts, only 7 (64%) impose a publication requirement and 5 (46%) offer journal clubs. Overall, 5 (45%) programs have onsite or on call clinical oversight of residents working in the emergency department by a specialist or senior doctor Table 2.

**Table 2:** Teaching and evaluation

Teaching components	Frequency
	n (%)
Lecture and Didactics (*N* = 11)	11 (100)
Lectures by residents (*N* = 11)	11 (100)
Journal clubs (*N* = 11)	5 (45.5)
Research requirement (*N* = 11)	11 (100)
Publication requirement (*N* = 11)	7 (63.6)
Clinical shifts supervision (*N* = 11)	5 (45.5)
Evaluation by examination (*N* = 11)	7 (63.6)

Five out of 10 programs have mandatory biomedical sciences courses during the first years of training. These include physiology, pharmacology, microbiology, epidemiology and biostatistics, and anatomy. Candidates are expected to pass these biomedical courses prior to completion of EM training.

### Clinical rotations and biomedical science courses during training

All EMRP offers clinical rotations in Emergency Medicine, Paediatrics, Obstetrics and Gynaecology. Internal Medicine and Orthopaedics and Traumatology clinical rotations are offered in 90% of programs. Six programs expose their residents to pre-hospital care or Emergency Medical Services (EMS). Only 20% of programs offer rotations in radiology and neonatal intensive care units Table 3.

**Table 3:** Clinical rotations during training

Clinical Rotation	Number of EMRP	Percentage
	*N* = 10	%
Emergency Medicine	10	100
Paediatrics and Child Health	10	100
Obstetrics and gynaecology	10	100
Internal medicine	9	90
Orthopedics and Trauma	9	90
Anesthesia	8	80
Medical intensive care unit	8	80
General Surgery	7	70
Pre-hospital medicine or EMS	6	60
Neurosurgery	5	50
Psychiatry and mental health	4	40
Surgical intensive care unit	4	40
Pediatrics intensive care unit	4	40
Toxicology	3	30
International visiting EM Experience	3	30
Radiology and Imaging	2	20
Neonatal Intensive care unit	2	20

### Emergency department clinical shifts

In all EMPR, when rotating in the Emergency Medicine department, residents cover a median of 16 clinical shifts in resident years one, two and four, and 15 clinical shifts in year 3. The average duration of a single clinical shift in each EMRP was found to be 12 h regardless of the year of training Table 4.

**Table 4:** Emergency Department clinical shifts

Academic year	Average number ED clinical shifts per month	
	Median (number)	IQR (number)
Resident Year 1	16	14–21
Resident Year 2	16	14–20
Resident Year 3	15	13–16
Resident Year 4*	16	14–17
Academic year	Average duration (in hours) of single ED clinical shift	
	Median (hours)	IQR (hours)
Resident Year 1	12	10.5–12
Resident Year 2	12	11–12
Resident Year 3	12	12–12
Resident Year 4*	12	10.8–12

*7/10 programs offers a 4 year EMRP

## Discussion

To the best of our knowledge, this is the first and most comprehensive report on the assessment of EMRP curricula structure and content in Africa. Although EM is on the rapid raise, both in terms of speciality training and recognition, less than one third of African countries have at least one EMRP. This poses a substantial challenge to the need to rapidly increase the number of EM experts who can take the lead in supporting the development of ECS across Africa.

In countries were the speciality of emergency medicine is well established, the initial programs of EM training were largely done by doctors and faculty with no background in Emergency medicine [11]. Similar to these observations, over 80 % of EMRPs in Africa were started by local faculty without EM training. Of note, one third of these programs were supported by international partners who partnered with local faculty to run these programs. Two programs were started exclusively by international partners. While our study did not focus on the sustainability of these programs, we believe having engaged local faculty champions involvinde from the beginning might increase the likelihood of successful EMRP implementation and help ensure sustainability once the local partners transition the programs to the local faculty. All the EMRPs in Africa share the same minimum entry criteria of having at least a doctor of medicine (MD) or equivalent and internship experience. This is a slight higher requirement for entry into an EM residency compared to some high-income countries [12], in which a candidate only needs to have MD, and the first year of residency is counted as internship year, regardless of the length of training. Nearly two thirds of the programs offer a 4-year EMRP curriculum, while the rest offer a 3-year program. We observed that the duration of EMRP training was dependent on two main factors: the first was the length of other local residency training programs, such as internal medicine and surgery; and the second was the the international partner that was the source of curriculum that influenced the programs. Most curricula were modelled from other established programs from across the world, and we noted that the content and rotations of such programs frequently matched most of the rotations in the countries after which the curricula were modelled. Interestingly, half of the EMRP curricula content were also influenced by local or international professional societies like AFEM. While this shows the important role of a professional society in a young and evolving speciality, it further opens the possibility of harmonization of training, paving the way for standardization of examination and the possibility of formimg regional accreditation bodies of emergency medicine in Africa. Most of the programs have reported changes to their curriculum within the last 5 years, with key changes including the incorporation of simulation training, integration of in-training assessment, and increased focus on management skills. We believe these changes might have been brought up by the growth and development of simulation technology in medical training and the market need for competency in clinical skills [13–15].

All EMRPs offer didactic sessions, and the residents are heavily involved in preparation and delivery of these sessions during their clinical training. This practice is similar to other EMRPs in high income countries (HIC) in which moderated didactics forms a core component of EMRP curricula [16, 17]. Half of the programs do not have journal club sessions, which might limit the capacity of residents to learn how to properly review and critique a scientific article [18]. Contrary to most training curricula from HIC [19], we found half of the EMRPs have a mandatory requirement that residents study and pass biomedical science subjects in the course of training. Furthermore, we found that all African EMRPs are research intensive, as they require all their candidates to conduct a research project during training and submit the product as a requirement for graduation. Furthermore, nearly two-third of the programs expect a candidate to publish the contents of the research conducted during residency training.

In addition to clinical rotation in the ED, all EMRPs expose their residents to paediatrics and obstetrics and gynaecology rotations. While this is similar to other well-established international EMRPs, we believe this provides the maximum benefit to decrease the burden of mortality and morbidity in Africa, as the largest burden of mortality is in emergency care (including injury), paediatrics population and maternal health [20]. Despite the large burden of poisoning from different sources in Africa [21, 22], less than one third of programs offer a rotation in toxicology. We believe this might be caused by number of factors including the lack of dedicated poison control centres in most African countries, and lack of speciality training in toxicology [23]. Of interest, six out of ten programs provide their residents with clinical exposure to pre-hospital or Emergency Medical Services rotations. This is promising as most of African countries represented in this study have no formal pre-hospital systems, and hence exposure, experience and training in pre-hospital care will help to develop local experts that can champion the evolution of formal out of hospital care systems.

Similar to most established EMRPs in the world, the residents in all programs cover an average of 12 h per each clinical shift [24]; however, less than half of programs offer in-ED clinical oversight of residents by specialist or senior faculty. In those with ED supervision, most of the specialists are either physically available during certain hours of the day or are available on call for consultation.

## Limitations

This was a non-validated online-based survey and despite our efforts to identify and invite participation by all known EMRPs, not all programs responded. However, we did receive responses from the majority of EMRPs that are representative of each of the geopolitical regions. The specific program data are the result of non-validated responses of the leadership of each EMRP; we couldn’t ascertain the identity of respondent and did not assess the non-response bias.

## Conclusion

Most EMRPs in Africa were started by non-EM trained local faculty alone or collaboration with international partners. The curricula offers most exposure to emergency medicine, paediatrics, and obstetrics and gynaecology, with less exposure to toxicology, radiology, and neonatal intensive care training. Residents are highly involved in leading didactics and journal clubs and fewer than one half of programs have in-ED specialist supervision of the clinical care delivered by the residents.

## Additional file

Additional file 1: Emergency Medicine Residency Training in Africa Survey Tool. (PDF 200 kb)

## Data Availability

The datasets used and/or analysed during the current study are available from the corresponding author on reasonable request.

## Abbreviations

AFEM: African Federation for Emergency Medicine
AFJEM: African Journal of Emergency Medicine
ECS: Emergency Care Systems
ED: Emergency Department
EM: Emergency Medicine
EMRP: Emergency Medicine Residency-training Programs
EMS: Emergency Medical Services
HIC: High Income Country
MD: Medical Doctor
USA: United States of America
