# Supplementary material for: Emergency medicine residency training in Africa: overview of curriculum
Source: BMC Med Educ. 2019 Jul 31;19:294. doi: 10.1186/s12909-019-1729-1 (PMC6670225; doi:10.1186/s12909-019-1729-1)
Supplement: Supplementary file 1 — Emergency Medicine Residency Training in Africa Survey Tool. (PDF 200 kb) [file 12909_2019_1729_MOESM1_ESM.pdf]

Emergency Medicine Residency Curriculum in Africa

Please complete the survey below.

Thank you!

General Residency Questions

What country is your Emergency Medicine program in? __________________________

What Universit(ies) is your Emergency Medicine program housed at? __________________________

When did your Emergency Medicine program start?

- ◯ 1980
- ◯ 1981
- ◯ 1982
- ◯ 1983
- ◯ 1984
- ◯ 1985
- ◯ 1986
- ◯ 1987
- ◯ 1988
- ◯ 1989
- ◯ 1990
- ◯ 1991
- ◯ 1992
- ◯ 1993
- ◯ 1994
- ◯ 1995
- ◯ 1996
- ◯ 1997
- ◯ 1998
- ◯ 1999
- ◯ 2000
- ◯ 2001
- ◯ 2002
- ◯ 2003
- ◯ 2004
- ◯ 2005
- ◯ 2006
- ◯ 2007
- ◯ 2008
- ◯ 2009
- ◯ 2010
- ◯ 2011
- ◯ 2012
- ◯ 2013
- ◯ 2014
- ◯ 2015
- ◯ 2016
- ◯ 2017

Is Emergency Medicine a recognized speciality in your country?

- ◯ Yes
- ◯ No

Do you have a board or college that accredits all EM graduates in your country?

- ◯ Yes
- ◯ No

What are the MINIMUM entry requirements for the Emergency Medicine residency program (must be MD, Intern, GPA cutoff, work experience, etc) ? __________________________________

What are the MINIMUM graduation requirements for Emergency Medicine program (must pass all course work, department recommendations, etc)? __________________________________

How long is your residency training (in years)

- ◯ 1
- ◯ 2
- ◯ 3
- ◯ 4
- ◯ 5
- ◯ 6
- ◯ 7
- ◯ 8
- ◯ 9
- ◯ 10

How many residents do you accept in your residency program(the actual number on average each year)?

◯ 0
◯ 1
◯ 2
◯ 3
◯ 4
◯ 5
◯ 6
◯ 7
◯ 8
◯ 9
◯ 10
◯ 11
◯ 12
◯ 13
◯ 14
◯ 15
◯ 16
◯ 17
◯ 18
◯ 19
◯ 20
◯ 21
◯ 22
◯ 23
◯ 24
◯ 25
◯ 26
◯ 27
◯ 28
◯ 29
◯ 30
◯ 31
◯ 32
◯ 33
◯ 34
◯ 35
◯ 36
◯ 37
◯ 38
◯ 39
◯ 40
◯ 41
◯ 42
◯ 43
◯ 44
◯ 45
◯ 46
◯ 47
◯ 48
◯ 49
◯ 50
◯ 51
◯ 52
◯ 53
◯ 54
◯ 55
◯ 56
◯ 57
◯ 58
◯ 59
◯ 60
◯ 61
◯ 62
◯ 63
◯ 64
◯ 65
◯ 66
◯ 67
◯ 68
◯ 69

- ◯ 70
- ◯ 71
- ◯ 72
- ◯ 73
- ◯ 74
- ◯ 75
- ◯ 76
- ◯ 77
- ◯ 78
- ◯ 79
- ◯ 80
- ◯ 81
- ◯ 82
- ◯ 83
- ◯ 84
- ◯ 85
- ◯ 86
- ◯ 87
- ◯ 88
- ◯ 89
- ◯ 90
- ◯ 91
- ◯ 92
- ◯ 93
- ◯ 94
- ◯ 95
- ◯ 96
- ◯ 97
- ◯ 98
- ◯ 99
- ◯ 100

What is the percentage of foreign students in your program (estimate of current prospective students for the entire program)

◯ 0
◯ 1
◯ 2
◯ 3
◯ 4
◯ 5
◯ 6
◯ 7
◯ 8
◯ 9
◯ 10
◯ 11
◯ 12
◯ 13
◯ 14
◯ 15
◯ 16
◯ 17
◯ 18
◯ 19
◯ 20
◯ 21
◯ 22
◯ 23
◯ 24
◯ 25
◯ 26
◯ 27
◯ 28
◯ 29
◯ 30
◯ 31
◯ 32
◯ 33
◯ 34
◯ 35
◯ 36
◯ 37
◯ 38
◯ 39
◯ 40
◯ 41
◯ 42
◯ 43
◯ 44
◯ 45
◯ 46
◯ 47
◯ 48
◯ 49
◯ 50
◯ 51
◯ 52
◯ 53
◯ 54
◯ 55
◯ 56
◯ 57
◯ 58
◯ 59
◯ 60
◯ 61
◯ 62
◯ 63
◯ 64
◯ 65
◯ 66
◯ 67
◯ 68
◯ 69

◯ 70
◯ 71
◯ 72
◯ 73
◯ 74
◯ 75
◯ 76
◯ 77
◯ 78
◯ 79
◯ 80
◯ 81
◯ 82
◯ 83
◯ 84
◯ 85
◯ 86
◯ 87
◯ 88
◯ 89
◯ 90
◯ 91
◯ 92
◯ 93
◯ 94
◯ 95
◯ 96
◯ 97
◯ 98
◯ 99
◯ 100

What is the overall pass rate (to graduation) in your program (estimate in percentage)

◯ 0
◯ 1
◯ 2
◯ 3
◯ 4
◯ 5
◯ 6
◯ 7
◯ 8
◯ 9
◯ 10
◯ 11
◯ 12
◯ 13
◯ 14
◯ 15
◯ 16
◯ 17
◯ 18
◯ 19
◯ 20
◯ 21
◯ 22
◯ 23
◯ 24
◯ 25
◯ 26
◯ 27
◯ 28
◯ 29
◯ 30
◯ 31
◯ 32
◯ 33
◯ 34
◯ 35
◯ 36
◯ 37
◯ 38
◯ 39
◯ 40
◯ 41
◯ 42
◯ 43
◯ 44
◯ 45
◯ 46
◯ 47
◯ 48
◯ 49
◯ 50
◯ 51
◯ 52
◯ 53
◯ 54
◯ 55
◯ 56
◯ 57
◯ 58
◯ 59
◯ 60
◯ 61
◯ 62
◯ 63
◯ 64
◯ 65
◯ 66
◯ 67
◯ 68
◯ 69

◯ 70
◯ 71
◯ 72
◯ 73
◯ 74
◯ 75
◯ 76
◯ 77
◯ 78
◯ 79
◯ 80
◯ 81
◯ 82
◯ 83
◯ 84
◯ 85
◯ 86
◯ 87
◯ 88
◯ 89
◯ 90
◯ 91
◯ 92
◯ 93
◯ 94
◯ 95
◯ 96
◯ 97
◯ 98
◯ 99
◯ 100

Do you have collaborations both intra and inter country? (exchange programs, visiting faculty..etc)

◯ Yes
◯ No

If yes, mention them.. ________________________________

Faculty related questions

How many full-time faculty (university employed) do you have interacting with residents (including in the ED and in didactics)

- ◯ 0
- ◯ 1
- ◯ 2
- ◯ 3
- ◯ 4
- ◯ 5
- ◯ 6
- ◯ 7
- ◯ 8
- ◯ 9
- ◯ 10
- ◯ 11
- ◯ 12
- ◯ 13
- ◯ 14
- ◯ 15
- ◯ 16
- ◯ 17
- ◯ 18
- ◯ 19
- ◯ 20
- ◯ 21
- ◯ 22
- ◯ 23
- ◯ 24
- ◯ 25
- ◯ 26
- ◯ 27
- ◯ 28
- ◯ 29
- ◯ 30
- ◯ 31
- ◯ 32
- ◯ 33
- ◯ 34
- ◯ 35
- ◯ 36
- ◯ 37
- ◯ 38
- ◯ 39
- ◯ 40
- ◯ 41
- ◯ 42
- ◯ 43
- ◯ 44
- ◯ 45
- ◯ 46
- ◯ 47
- ◯ 48
- ◯ 49
- ◯ 50
- ◯ 51
- ◯ 52
- ◯ 53
- ◯ 54
- ◯ 55
- ◯ 56
- ◯ 57
- ◯ 58
- ◯ 59
- ◯ 60
- ◯ 61
- ◯ 62
- ◯ 63
- ◯ 64
- ◯ 65
- ◯ 66
- ◯ 67
- ◯ 68
- ◯ 69

- ◯ 70
- ◯ 71
- ◯ 72
- ◯ 73
- ◯ 74
- ◯ 75
- ◯ 76
- ◯ 77
- ◯ 78
- ◯ 79
- ◯ 80
- ◯ 81
- ◯ 82
- ◯ 83
- ◯ 84
- ◯ 85
- ◯ 86
- ◯ 87
- ◯ 88
- ◯ 89
- ◯ 90
- ◯ 91
- ◯ 92
- ◯ 93
- ◯ 94
- ◯ 95
- ◯ 96
- ◯ 97
- ◯ 98
- ◯ 99
- ◯ 100

How many of these full-time faculty (university employed) are men?

- ◯ 0
- ◯ 1
- ◯ 2
- ◯ 3
- ◯ 4
- ◯ 5
- ◯ 6
- ◯ 7
- ◯ 8
- ◯ 9
- ◯ 10
- ◯ 11
- ◯ 12
- ◯ 13
- ◯ 14
- ◯ 15
- ◯ 16
- ◯ 17
- ◯ 18
- ◯ 19
- ◯ 20
- ◯ 21
- ◯ 22
- ◯ 23
- ◯ 24
- ◯ 25
- ◯ 26
- ◯ 27
- ◯ 28
- ◯ 29
- ◯ 30
- ◯ 31
- ◯ 32
- ◯ 33
- ◯ 34
- ◯ 35
- ◯ 36
- ◯ 37
- ◯ 38
- ◯ 39
- ◯ 40
- ◯ 41
- ◯ 42
- ◯ 43
- ◯ 44
- ◯ 45
- ◯ 46
- ◯ 47
- ◯ 48
- ◯ 49
- ◯ 50
- ◯ 51
- ◯ 52
- ◯ 53
- ◯ 54
- ◯ 55
- ◯ 56
- ◯ 57
- ◯ 58
- ◯ 59
- ◯ 60
- ◯ 61
- ◯ 62
- ◯ 63
- ◯ 64
- ◯ 65
- ◯ 66
- ◯ 67
- ◯ 68
- ◯ 69

◯ 70
◯ 71
◯ 72
◯ 73
◯ 74
◯ 75
◯ 76
◯ 77
◯ 78
◯ 79
◯ 80
◯ 81
◯ 82
◯ 83
◯ 84
◯ 85
◯ 86
◯ 87
◯ 88
◯ 89
◯ 90
◯ 91
◯ 92
◯ 93
◯ 94
◯ 95
◯ 96
◯ 97
◯ 98
◯ 99
◯ 100

How many part_time or honorary faculty do you have interacting with residents (including in the ED and in didactics)

- ◯ 0
- ◯ 1
- ◯ 2
- ◯ 3
- ◯ 4
- ◯ 5
- ◯ 6
- ◯ 7
- ◯ 8
- ◯ 9
- ◯ 10
- ◯ 11
- ◯ 12
- ◯ 13
- ◯ 14
- ◯ 15
- ◯ 16
- ◯ 17
- ◯ 18
- ◯ 19
- ◯ 20
- ◯ 21
- ◯ 22
- ◯ 23
- ◯ 24
- ◯ 25
- ◯ 26
- ◯ 27
- ◯ 28
- ◯ 29
- ◯ 30
- ◯ 31
- ◯ 32
- ◯ 33
- ◯ 34
- ◯ 35
- ◯ 36
- ◯ 37
- ◯ 38
- ◯ 39
- ◯ 40
- ◯ 41
- ◯ 42
- ◯ 43
- ◯ 44
- ◯ 45
- ◯ 46
- ◯ 47
- ◯ 48
- ◯ 49
- ◯ 50
- ◯ 51
- ◯ 52
- ◯ 53
- ◯ 54
- ◯ 55
- ◯ 56
- ◯ 57
- ◯ 58
- ◯ 59
- ◯ 60
- ◯ 61
- ◯ 62
- ◯ 63
- ◯ 64
- ◯ 65
- ◯ 66
- ◯ 67
- ◯ 68
- ◯ 69

◯ 70
◯ 71
◯ 72
◯ 73
◯ 74
◯ 75
◯ 76
◯ 77
◯ 78
◯ 79
◯ 80
◯ 81
◯ 82
◯ 83
◯ 84
◯ 85
◯ 86
◯ 87
◯ 88
◯ 89
◯ 90
◯ 91
◯ 92
◯ 93
◯ 94
◯ 95
◯ 96
◯ 97
◯ 98
◯ 99
◯ 100

How many of these part_time faculty (honorary) are foreigners?

- ◯ 0
- ◯ 1
- ◯ 2
- ◯ 3
- ◯ 4
- ◯ 5
- ◯ 6
- ◯ 7
- ◯ 8
- ◯ 9
- ◯ 10
- ◯ 11
- ◯ 12
- ◯ 13
- ◯ 14
- ◯ 15
- ◯ 16
- ◯ 17
- ◯ 18
- ◯ 19
- ◯ 20
- ◯ 21
- ◯ 22
- ◯ 23
- ◯ 24
- ◯ 25
- ◯ 26
- ◯ 27
- ◯ 28
- ◯ 29
- ◯ 30
- ◯ 31
- ◯ 32
- ◯ 33
- ◯ 34
- ◯ 35
- ◯ 36
- ◯ 37
- ◯ 38
- ◯ 39
- ◯ 40
- ◯ 41
- ◯ 42
- ◯ 43
- ◯ 44
- ◯ 45
- ◯ 46
- ◯ 47
- ◯ 48
- ◯ 49
- ◯ 50
- ◯ 51
- ◯ 52
- ◯ 53
- ◯ 54
- ◯ 55
- ◯ 56
- ◯ 57
- ◯ 58
- ◯ 59
- ◯ 60
- ◯ 61
- ◯ 62
- ◯ 63
- ◯ 64
- ◯ 65
- ◯ 66
- ◯ 67
- ◯ 68
- ◯ 69

- ◯ 70
- ◯ 71
- ◯ 72
- ◯ 73
- ◯ 74
- ◯ 75
- ◯ 76
- ◯ 77
- ◯ 78
- ◯ 79
- ◯ 80
- ◯ 81
- ◯ 82
- ◯ 83
- ◯ 84
- ◯ 85
- ◯ 86
- ◯ 87
- ◯ 88
- ◯ 89
- ◯ 90
- ◯ 91
- ◯ 92
- ◯ 93
- ◯ 94
- ◯ 95
- ◯ 96
- ◯ 97
- ◯ 98
- ◯ 99
- ◯ 100

How many of these part_time faculty (honorary) are men?

◯ 0
◯ 1
◯ 2
◯ 3
◯ 4
◯ 5
◯ 6
◯ 7
◯ 8
◯ 9
◯ 10
◯ 11
◯ 12
◯ 13
◯ 14
◯ 15
◯ 16
◯ 17
◯ 18
◯ 19
◯ 20
◯ 21
◯ 22
◯ 23
◯ 24
◯ 25
◯ 26
◯ 27
◯ 28
◯ 29
◯ 30
◯ 31
◯ 32
◯ 33
◯ 34
◯ 35
◯ 36
◯ 37
◯ 38
◯ 39
◯ 40
◯ 41
◯ 42
◯ 43
◯ 44
◯ 45
◯ 46
◯ 47
◯ 48
◯ 49
◯ 50
◯ 51
◯ 52
◯ 53
◯ 54
◯ 55
◯ 56
◯ 57
◯ 58
◯ 59
◯ 60
◯ 61
◯ 62
◯ 63
◯ 64
◯ 65
◯ 66
◯ 67
◯ 68
◯ 69

◯ 70
◯ 71
◯ 72
◯ 73
◯ 74
◯ 75
◯ 76
◯ 77
◯ 78
◯ 79
◯ 80
◯ 81
◯ 82
◯ 83
◯ 84
◯ 85
◯ 86
◯ 87
◯ 88
◯ 89
◯ 90
◯ 91
◯ 92
◯ 93
◯ 94
◯ 95
◯ 96
◯ 97
◯ 98
◯ 99
◯ 100

What are the specialty qualifications/training of the faculty?

◯ Emergency Medicine specialist or super specialists only
◯ Emergency Medicine and other non EM specialist
◯ Other non EM specialist
◯ Others

Mention other qualifications ________________________________

How many faculty (university employed and honorary) are qualified as emergency medicine specialists?

- ◯ 0
- ◯ 1
- ◯ 2
- ◯ 3
- ◯ 4
- ◯ 5
- ◯ 6
- ◯ 7
- ◯ 8
- ◯ 9
- ◯ 10
- ◯ 11
- ◯ 12
- ◯ 13
- ◯ 14
- ◯ 15
- ◯ 16
- ◯ 17
- ◯ 18
- ◯ 19
- ◯ 20
- ◯ 21
- ◯ 22
- ◯ 23
- ◯ 24
- ◯ 25
- ◯ 26
- ◯ 27
- ◯ 28
- ◯ 29
- ◯ 30
- ◯ 31
- ◯ 32
- ◯ 33
- ◯ 34
- ◯ 35
- ◯ 36
- ◯ 37
- ◯ 38
- ◯ 39
- ◯ 40
- ◯ 41
- ◯ 42
- ◯ 43
- ◯ 44
- ◯ 45
- ◯ 46
- ◯ 47
- ◯ 48
- ◯ 49
- ◯ 50
- ◯ 51
- ◯ 52
- ◯ 53
- ◯ 54
- ◯ 55
- ◯ 56
- ◯ 57
- ◯ 58
- ◯ 59
- ◯ 60
- ◯ 61
- ◯ 62
- ◯ 63
- ◯ 64
- ◯ 65
- ◯ 66
- ◯ 67
- ◯ 68
- ◯ 69

◯ 70
◯ 71
◯ 72
◯ 73
◯ 74
◯ 75
◯ 76
◯ 77
◯ 78
◯ 79
◯ 80
◯ 81
◯ 82
◯ 83
◯ 84
◯ 85
◯ 86
◯ 87
◯ 88
◯ 89
◯ 90
◯ 91
◯ 92
◯ 93
◯ 94
◯ 95
◯ 96
◯ 97
◯ 98
◯ 99
◯ 100

How many of these qualified emergency physicians were trained in your country? (estimates)

◯ 0
◯ 1
◯ 2
◯ 3
◯ 4
◯ 5
◯ 6
◯ 7
◯ 8
◯ 9
◯ 10
◯ 11
◯ 12
◯ 13
◯ 14
◯ 15
◯ 16
◯ 17
◯ 18
◯ 19
◯ 20
◯ 21
◯ 22
◯ 23
◯ 24
◯ 25
◯ 26
◯ 27
◯ 28
◯ 29
◯ 30
◯ 31
◯ 32
◯ 33
◯ 34
◯ 35
◯ 36
◯ 37
◯ 38
◯ 39
◯ 40
◯ 41
◯ 42
◯ 43
◯ 44
◯ 45
◯ 46
◯ 47
◯ 48
◯ 49
◯ 50
◯ 51
◯ 52
◯ 53
◯ 54
◯ 55
◯ 56
◯ 57
◯ 58
◯ 59
◯ 60
◯ 61
◯ 62
◯ 63
◯ 64
◯ 65
◯ 66
◯ 67
◯ 68
◯ 69

- ◯ 70
- ◯ 71
- ◯ 72
- ◯ 73
- ◯ 74
- ◯ 75
- ◯ 76
- ◯ 77
- ◯ 78
- ◯ 79
- ◯ 80
- ◯ 81
- ◯ 82
- ◯ 83
- ◯ 84
- ◯ 85
- ◯ 86
- ◯ 87
- ◯ 88
- ◯ 89
- ◯ 90
- ◯ 91
- ◯ 92
- ◯ 93
- ◯ 94
- ◯ 95
- ◯ 96
- ◯ 97
- ◯ 98
- ◯ 99
- ◯ 100

How many of these qualified emergency physicians were trained from your current program? (estimates)

○ 0
○ 1
○ 2
○ 3
○ 4
○ 5
○ 6
○ 7
○ 8
○ 9
○ 10
○ 11
○ 12
○ 13
○ 14
○ 15
○ 16
○ 17
○ 18
○ 19
○ 20
○ 21
○ 22
○ 23
○ 24
○ 25
○ 26
○ 27
○ 28
○ 29
○ 30
○ 31
○ 32
○ 33
○ 34
○ 35
○ 36
○ 37
○ 38
○ 39
○ 40
○ 41
○ 42
○ 43
○ 44
○ 45
○ 46
○ 47
○ 48
○ 49
○ 50
○ 51
○ 52
○ 53
○ 54
○ 55
○ 56
○ 57
○ 58
○ 59
○ 60
○ 61
○ 62
○ 63
○ 64
○ 65
○ 66
○ 67
○ 68
○ 69

◯ 70
◯ 71
◯ 72
◯ 73
◯ 74
◯ 75
◯ 76
◯ 77
◯ 78
◯ 79
◯ 80
◯ 81
◯ 82
◯ 83
◯ 84
◯ 85
◯ 86
◯ 87
◯ 88
◯ 89
◯ 90
◯ 91
◯ 92
◯ 93
◯ 94
◯ 95
◯ 96
◯ 97
◯ 98
◯ 99
◯ 100

Sustainability/ future of program questions

How many graduates do you have to date (since the program started)?

- 0
- 1
- 2
- 3
- 4
- 5
- 6
- 7
- 8
- 9
- 10
- 11
- 12
- 13
- 14
- 15
- 16
- 17
- 18
- 19
- 20
- 21
- 22
- 23
- 24
- 25
- 26
- 27
- 28
- 29
- 30
- 31
- 32
- 33
- 34
- 35
- 36
- 37
- 38
- 39
- 40
- 41
- 42
- 43
- 44
- 45
- 46
- 47
- 48
- 49
- 50
- 51
- 52
- 53
- 54
- 55
- 56
- 57
- 58
- 59
- 60
- 61
- 62
- 63
- 64
- 65
- 66
- 67
- 68
- 69

◯ 70
◯ 71
◯ 72
◯ 73
◯ 74
◯ 75
◯ 76
◯ 77
◯ 78
◯ 79
◯ 80
◯ 81
◯ 82
◯ 83
◯ 84
◯ 85
◯ 86
◯ 87
◯ 88
◯ 89
◯ 90
◯ 91
◯ 92
◯ 93
◯ 94
◯ 95
◯ 96
◯ 97
◯ 98
◯ 99
◯ 100

How many of these graduates were men?

- ◯ 0
- ◯ 1
- ◯ 2
- ◯ 3
- ◯ 4
- ◯ 5
- ◯ 6
- ◯ 7
- ◯ 8
- ◯ 9
- ◯ 10
- ◯ 11
- ◯ 12
- ◯ 13
- ◯ 14
- ◯ 15
- ◯ 16
- ◯ 17
- ◯ 18
- ◯ 19
- ◯ 20
- ◯ 21
- ◯ 22
- ◯ 23
- ◯ 24
- ◯ 25
- ◯ 26
- ◯ 27
- ◯ 28
- ◯ 29
- ◯ 30
- ◯ 31
- ◯ 32
- ◯ 33
- ◯ 34
- ◯ 35
- ◯ 36
- ◯ 37
- ◯ 38
- ◯ 39
- ◯ 40
- ◯ 41
- ◯ 42
- ◯ 43
- ◯ 44
- ◯ 45
- ◯ 46
- ◯ 47
- ◯ 48
- ◯ 49
- ◯ 50
- ◯ 51
- ◯ 52
- ◯ 53
- ◯ 54
- ◯ 55
- ◯ 56
- ◯ 57
- ◯ 58
- ◯ 59
- ◯ 60
- ◯ 61
- ◯ 62
- ◯ 63
- ◯ 64
- ◯ 65
- ◯ 66
- ◯ 67
- ◯ 68
- ◯ 69

○ 70
○ 71
○ 72
○ 73
○ 74
○ 75
○ 76
○ 77
○ 78
○ 79
○ 80
○ 81
○ 82
○ 83
○ 84
○ 85
○ 86
○ 87
○ 88
○ 89
○ 90
○ 91
○ 92
○ 93
○ 94
○ 95
○ 96
○ 97
○ 98
○ 99
○ 100

What percentage of your graduates stay as faculty within your program after graduation (estimates)?

○ 0
○ 1
○ 2
○ 3
○ 4
○ 5
○ 6
○ 7
○ 8
○ 9
○ 10
○ 11
○ 12
○ 13
○ 14
○ 15
○ 16
○ 17
○ 18
○ 19
○ 20
○ 21
○ 22
○ 23
○ 24
○ 25
○ 26
○ 27
○ 28
○ 29
○ 30
○ 31
○ 32
○ 33
○ 34
○ 35
○ 36
○ 37
○ 38
○ 39
○ 40
○ 41
○ 42
○ 43
○ 44
○ 45
○ 46
○ 47
○ 48
○ 49
○ 50
○ 51
○ 52
○ 53
○ 54
○ 55
○ 56
○ 57
○ 58
○ 59
○ 60
○ 61
○ 62
○ 63
○ 64
○ 65
○ 66
○ 67
○ 68
○ 69

◯ 70
◯ 71
◯ 72
◯ 73
◯ 74
◯ 75
◯ 76
◯ 77
◯ 78
◯ 79
◯ 80
◯ 81
◯ 82
◯ 83
◯ 84
◯ 85
◯ 86
◯ 87
◯ 88
◯ 89
◯ 90
◯ 91
◯ 92
◯ 93
◯ 94
◯ 95
◯ 96
◯ 97
◯ 98
◯ 99
◯ 100

What percentage of your graduates who are citizens of your country, have left to pursue EM jobs abroad after graduation (estimates)?

- ◯ 0
- ◯ 1
- ◯ 2
- ◯ 3
- ◯ 4
- ◯ 5
- ◯ 6
- ◯ 7
- ◯ 8
- ◯ 9
- ◯ 10
- ◯ 11
- ◯ 12
- ◯ 13
- ◯ 14
- ◯ 15
- ◯ 16
- ◯ 17
- ◯ 18
- ◯ 19
- ◯ 20
- ◯ 21
- ◯ 22
- ◯ 23
- ◯ 24
- ◯ 25
- ◯ 26
- ◯ 27
- ◯ 28
- ◯ 29
- ◯ 30
- ◯ 31
- ◯ 32
- ◯ 33
- ◯ 34
- ◯ 35
- ◯ 36
- ◯ 37
- ◯ 38
- ◯ 39
- ◯ 40
- ◯ 41
- ◯ 42
- ◯ 43
- ◯ 44
- ◯ 45
- ◯ 46
- ◯ 47
- ◯ 48
- ◯ 49
- ◯ 50
- ◯ 51
- ◯ 52
- ◯ 53
- ◯ 54
- ◯ 55
- ◯ 56
- ◯ 57
- ◯ 58
- ◯ 59
- ◯ 60
- ◯ 61
- ◯ 62
- ◯ 63
- ◯ 64
- ◯ 65
- ◯ 66
- ◯ 67
- ◯ 68
- ◯ 69

- ◯ 70
- ◯ 71
- ◯ 72
- ◯ 73
- ◯ 74
- ◯ 75
- ◯ 76
- ◯ 77
- ◯ 78
- ◯ 79
- ◯ 80
- ◯ 81
- ◯ 82
- ◯ 83
- ◯ 84
- ◯ 85
- ◯ 86
- ◯ 87
- ◯ 88
- ◯ 89
- ◯ 90
- ◯ 91
- ◯ 92
- ◯ 93
- ◯ 94
- ◯ 95
- ◯ 96
- ◯ 97
- ◯ 98
- ◯ 99
- ◯ 100

What percentage of your graduates who are citizens of your country, have gone-on to develop/staff emergency and casuality units in other regions/provinces within your country after graduation (estimates)?

- ◯ 0
- ◯ 1
- ◯ 2
- ◯ 3
- ◯ 4
- ◯ 5
- ◯ 6
- ◯ 7
- ◯ 8
- ◯ 9
- ◯ 10
- ◯ 11
- ◯ 12
- ◯ 13
- ◯ 14
- ◯ 15
- ◯ 16
- ◯ 17
- ◯ 18
- ◯ 19
- ◯ 20
- ◯ 21
- ◯ 22
- ◯ 23
- ◯ 24
- ◯ 25
- ◯ 26
- ◯ 27
- ◯ 28
- ◯ 29
- ◯ 30
- ◯ 31
- ◯ 32
- ◯ 33
- ◯ 34
- ◯ 35
- ◯ 36
- ◯ 37
- ◯ 38
- ◯ 39
- ◯ 40
- ◯ 41
- ◯ 42
- ◯ 43
- ◯ 44
- ◯ 45
- ◯ 46
- ◯ 47
- ◯ 48
- ◯ 49
- ◯ 50
- ◯ 51
- ◯ 52
- ◯ 53
- ◯ 54
- ◯ 55
- ◯ 56
- ◯ 57
- ◯ 58
- ◯ 59
- ◯ 60
- ◯ 61
- ◯ 62
- ◯ 63
- ◯ 64
- ◯ 65
- ◯ 66
- ◯ 67
- ◯ 68
- ◯ 69

- ◯ 70
- ◯ 71
- ◯ 72
- ◯ 73
- ◯ 74
- ◯ 75
- ◯ 76
- ◯ 77
- ◯ 78
- ◯ 79
- ◯ 80
- ◯ 81
- ◯ 82
- ◯ 83
- ◯ 84
- ◯ 85
- ◯ 86
- ◯ 87
- ◯ 88
- ◯ 89
- ◯ 90
- ◯ 91
- ◯ 92
- ◯ 93
- ◯ 94
- ◯ 95
- ◯ 96
- ◯ 97
- ◯ 98
- ◯ 99
- ◯ 100

What percentage of your graduates who are citizens of your country, have gone on to private practise within the country after graduation (estimates)?

○ 0
○ 1
○ 2
○ 3
○ 4
○ 5
○ 6
○ 7
○ 8
○ 9
○ 10
○ 11
○ 12
○ 13
○ 14
○ 15
○ 16
○ 17
○ 18
○ 19
○ 20
○ 21
○ 22
○ 23
○ 24
○ 25
○ 26
○ 27
○ 28
○ 29
○ 30
○ 31
○ 32
○ 33
○ 34
○ 35
○ 36
○ 37
○ 38
○ 39
○ 40
○ 41
○ 42
○ 43
○ 44
○ 45
○ 46
○ 47
○ 48
○ 49
○ 50
○ 51
○ 52
○ 53
○ 54
○ 55
○ 56
○ 57
○ 58
○ 59
○ 60
○ 61
○ 62
○ 63
○ 64
○ 65
○ 66
○ 67
○ 68
○ 69

- ◯ 70
- ◯ 71
- ◯ 72
- ◯ 73
- ◯ 74
- ◯ 75
- ◯ 76
- ◯ 77
- ◯ 78
- ◯ 79
- ◯ 80
- ◯ 81
- ◯ 82
- ◯ 83
- ◯ 84
- ◯ 85
- ◯ 86
- ◯ 87
- ◯ 88
- ◯ 89
- ◯ 90
- ◯ 91
- ◯ 92
- ◯ 93
- ◯ 94
- ◯ 95
- ◯ 96
- ◯ 97
- ◯ 98
- ◯ 99
- ◯ 100

What percentage of your graduates have left to pursue Non-EM related jobs /opportunities after graduation? (estimates)?

◯ 0
◯ 1
◯ 2
◯ 3
◯ 4
◯ 5
◯ 6
◯ 7
◯ 8
◯ 9
◯ 10
◯ 11
◯ 12
◯ 13
◯ 14
◯ 15
◯ 16
◯ 17
◯ 18
◯ 19
◯ 20
◯ 21
◯ 22
◯ 23
◯ 24
◯ 25
◯ 26
◯ 27
◯ 28
◯ 29
◯ 30
◯ 31
◯ 32
◯ 33
◯ 34
◯ 35
◯ 36
◯ 37
◯ 38
◯ 39
◯ 40
◯ 41
◯ 42
◯ 43
◯ 44
◯ 45
◯ 46
◯ 47
◯ 48
◯ 49
◯ 50
◯ 51
◯ 52
◯ 53
◯ 54
◯ 55
◯ 56
◯ 57
◯ 58
◯ 59
◯ 60
◯ 61
◯ 62
◯ 63
◯ 64
◯ 65
◯ 66
◯ 67
◯ 68
◯ 69

◯ 70
◯ 71
◯ 72
◯ 73
◯ 74
◯ 75
◯ 76
◯ 77
◯ 78
◯ 79
◯ 80
◯ 81
◯ 82
◯ 83
◯ 84
◯ 85
◯ 86
◯ 87
◯ 88
◯ 89
◯ 90
◯ 91
◯ 92
◯ 93
◯ 94
◯ 95
◯ 96
◯ 97
◯ 98
◯ 99
◯ 100

What is the plan for sustainability of your training program and for establishing EM as a financially viable career option?

____

Curriculum structure questions

Did you participate in the initial design of the curriculum and objectives in your institution?

◯ Yes
◯ No

If you participated in designing your curriculum, did you encounter any challenges

◯ Yes
◯ No
◯ Not applicable

Briefly explain the challenges you encountered.

____

EM training in your country was started by

◯ Local faculty who is EM trained
◯ Local faculty who is NOT-EM trained
◯ Local faculty who is EM trained in collaboration with International partners
◯ Local faculty who is NOT EM trained in collaboration with International partners
◯ International partners
◯ Others

mention other

____

Was the initial curriculum developed by EM trained Specialist ?

◯ Yes
◯ No

Is your curriculum modeled after another African, Australian, American, Asian or European program?
◯ Yes
◯ No

Which country was your curriculum modeled from? __________________________________

Does AFEM or IFEM or ACEP or SAEM or ACEM or Your National professional Society influence your curriculum?
◯ Yes
◯ No

Briefly elaborate how __________________________________

Has your curriculum changed since start of program?
◯ Yes
◯ No

Point out the key areas on the changes __________________________________

How often is the curriculum reviewed for changes?
◯ Once every 1 year
◯ Once every 2 years
◯ Once every 3 years
◯ Once every 4 years
◯ Once every 5 years
◯ Once every 10 years
◯ Once every 20 years
◯ Others
◯ No specific timing of review

Explain other __________________________________

Do you have any challenges implementing the current curriculum?
◯ Yes
◯ No

Explain the challenges __________________________________

Is your residency program a '3 years' program?
◯ Yes
◯ No

Below are questions regarding the rotations that your residents perform.
Please indicate which rotations your residents perform during first year through fourth year plus.

	Residency year 1	Residency year 2	Residency year 3	Residency year 4	Residency year 4+
Emergency Medicine	☐	☐	☐	☐	☐
Internal medicine	☐	☐	☐	☐	☐
Pediatrics	☐	☐	☐	☐	☐
Cardiology	☐	☐	☐	☐	☐
Obstetrics and gynaecology	☐	☐	☐	☐	☐
Orthopedics and Trauma	☐	☐	☐	☐	☐
Surgery	☐	☐	☐	☐	☐
Neurosurgery	☐	☐	☐	☐	☐
Anesthesia	☐	☐	☐	☐	☐
Radiology	☐	☐	☐	☐	☐
Psychiatry	☐	☐	☐	☐	☐
Toxicology	☐	☐	☐	☐	☐
Elective	☐	☐	☐	☐	☐
Pre-hospital medicine/EMS	☐	☐	☐	☐	☐
Medical intensive care unit	☐	☐	☐	☐	☐
Pediatrics intensive care unit	☐	☐	☐	☐	☐
Neonatal Intensive care unit	☐	☐	☐	☐	☐
Surgical intensive care unit	☐	☐	☐	☐	☐
Cardiac intensive care unit	☐	☐	☐	☐	☐
Neurologic intensive care unit	☐	☐	☐	☐	☐
International visiting elective(countries and programs?)	☐	☐	☐	☐	☐
other	☐	☐	☐	☐	☐
No external rotation	☐	☐	☐	☐	☐
Not applicable	☐	☐	☐	☐	☐

Briefly mention duration in weeks for rotations offered to year one residents per each rotation. __________________________

Briefly mention duration in weeks for rotations offered to year two residents per each rotation. __________________________

Briefly mention duration in weeks for rotations offered to year three residents per each rotation __________________________

Briefly mention duration in weeks for rotations offered to year four residents per each rotation __________________________

Briefly mention duration in weeks for rotations offered to year four + residents per each rotation.

____

Does your residency training program include mandatory biomedical science courses (example anatomy, physiology, microbiology, pharmacology etc)?

◯ Yes
◯ No

List the biomedical courses that your residents undertake

____

When rotating in Emergency Medicine department ,how many shifts per month do your 1st year residents cover within the emergency department?(average)

〇 1
〇 2
〇 3
〇 4
〇 5
〇 6
〇 7
〇 8
〇 9
〇 10
〇 11
〇 12
〇 13
〇 14
〇 15
〇 16
〇 17
〇 18
〇 19
〇 20
〇 21
〇 22
〇 23
〇 24
〇 25
〇 26
〇 27
〇 28
〇 29
〇 30
〇 31
〇 32
〇 33
〇 34
〇 35
〇 36
〇 37
〇 38
〇 39
〇 40
〇 41
〇 42
〇 43
〇 44
〇 45
〇 46
〇 47
〇 48
〇 49
〇 50
〇 51
〇 52
〇 53
〇 54
〇 55
〇 56
〇 57
〇 58
〇 59
〇 60

When rotating in Emergency Medicine department ,how many shifts per month do your 2nd year residents cover within the emergency department?(average)

- ◯ 1
- ◯ 2
- ◯ 3
- ◯ 4
- ◯ 5
- ◯ 6
- ◯ 7
- ◯ 8
- ◯ 9
- ◯ 10
- ◯ 11
- ◯ 12
- ◯ 13
- ◯ 14
- ◯ 15
- ◯ 16
- ◯ 17
- ◯ 18
- ◯ 19
- ◯ 20
- ◯ 21
- ◯ 22
- ◯ 23
- ◯ 24
- ◯ 25
- ◯ 26
- ◯ 27
- ◯ 28
- ◯ 29
- ◯ 30
- ◯ 31
- ◯ 32
- ◯ 33
- ◯ 34
- ◯ 35
- ◯ 36
- ◯ 37
- ◯ 38
- ◯ 39
- ◯ 40
- ◯ 41
- ◯ 42
- ◯ 43
- ◯ 44
- ◯ 45
- ◯ 46
- ◯ 47
- ◯ 48
- ◯ 49
- ◯ 50
- ◯ 51
- ◯ 52
- ◯ 53
- ◯ 54
- ◯ 55
- ◯ 56
- ◯ 57
- ◯ 58
- ◯ 59
- ◯ 60

When rotating in Emergency Medicine department,how many shifts per month do your 3rd year residents cover within the emergency department?(average)

- ◯ 1
- ◯ 2
- ◯ 3
- ◯ 4
- ◯ 5
- ◯ 6
- ◯ 7
- ◯ 8
- ◯ 9
- ◯ 10
- ◯ 11
- ◯ 12
- ◯ 13
- ◯ 14
- ◯ 15
- ◯ 16
- ◯ 17
- ◯ 18
- ◯ 19
- ◯ 20
- ◯ 21
- ◯ 22
- ◯ 23
- ◯ 24
- ◯ 25
- ◯ 26
- ◯ 27
- ◯ 28
- ◯ 29
- ◯ 30
- ◯ 31
- ◯ 32
- ◯ 33
- ◯ 34
- ◯ 35
- ◯ 36
- ◯ 37
- ◯ 38
- ◯ 39
- ◯ 40
- ◯ 41
- ◯ 42
- ◯ 43
- ◯ 44
- ◯ 45
- ◯ 46
- ◯ 47
- ◯ 48
- ◯ 49
- ◯ 50
- ◯ 51
- ◯ 52
- ◯ 53
- ◯ 54
- ◯ 55
- ◯ 56
- ◯ 57
- ◯ 58
- ◯ 59
- ◯ 60

When rotating in Emergency Medicine department,how many shifts per month do your 4th year residents cover within the emergency department?(average)

◯ 1
◯ 2
◯ 3
◯ 4
◯ 5
◯ 6
◯ 7
◯ 8
◯ 9
◯ 10
◯ 11
◯ 12
◯ 13
◯ 14
◯ 15
◯ 16
◯ 17
◯ 18
◯ 19
◯ 20
◯ 21
◯ 22
◯ 23
◯ 24
◯ 25
◯ 26
◯ 27
◯ 28
◯ 29
◯ 30
◯ 31
◯ 32
◯ 33
◯ 34
◯ 35
◯ 36
◯ 37
◯ 38
◯ 39
◯ 40
◯ 41
◯ 42
◯ 43
◯ 44
◯ 45
◯ 46
◯ 47
◯ 48
◯ 49
◯ 50
◯ 51
◯ 52
◯ 53
◯ 54
◯ 55
◯ 56
◯ 57
◯ 58
◯ 59
◯ 60

When rotating in Emergency Medicine department,how many shifts per month do your 4+ year residents cover within the emergency department? (average)

- ◯ 1
- ◯ 2
- ◯ 3
- ◯ 4
- ◯ 5
- ◯ 6
- ◯ 7
- ◯ 8
- ◯ 9
- ◯ 10
- ◯ 11
- ◯ 12
- ◯ 13
- ◯ 14
- ◯ 15
- ◯ 16
- ◯ 17
- ◯ 18
- ◯ 19
- ◯ 20
- ◯ 21
- ◯ 22
- ◯ 23
- ◯ 24
- ◯ 25
- ◯ 26
- ◯ 27
- ◯ 28
- ◯ 29
- ◯ 30
- ◯ 31
- ◯ 32
- ◯ 33
- ◯ 34
- ◯ 35
- ◯ 36
- ◯ 37
- ◯ 38
- ◯ 39
- ◯ 40
- ◯ 41
- ◯ 42
- ◯ 43
- ◯ 44
- ◯ 45
- ◯ 46
- ◯ 47
- ◯ 48
- ◯ 49
- ◯ 50
- ◯ 51
- ◯ 52
- ◯ 53
- ◯ 54
- ◯ 55
- ◯ 56
- ◯ 57
- ◯ 58
- ◯ 59
- ◯ 60

How many hours is the shifts for your 1st year residents?

◯ 1
◯ 2
◯ 3
◯ 4
◯ 5
◯ 6
◯ 7
◯ 8
◯ 9
◯ 10
◯ 11
◯ 12
◯ 13
◯ 14
◯ 15
◯ 16
◯ 17
◯ 18
◯ 19
◯ 20
◯ 21
◯ 22
◯ 23
◯ 24

How many hours is the shifts for your 2nd year residents?

◯ 1
◯ 2
◯ 3
◯ 4
◯ 5
◯ 6
◯ 7
◯ 8
◯ 9
◯ 10
◯ 11
◯ 12
◯ 13
◯ 14
◯ 15
◯ 16
◯ 17
◯ 18
◯ 19
◯ 20
◯ 21
◯ 22
◯ 23
◯ 24

How many hours is the shifts for your 3rd year residents?

- ◯ 1
- ◯ 2
- ◯ 3
- ◯ 4
- ◯ 5
- ◯ 6
- ◯ 7
- ◯ 8
- ◯ 9
- ◯ 10
- ◯ 11
- ◯ 12
- ◯ 13
- ◯ 14
- ◯ 15
- ◯ 16
- ◯ 17
- ◯ 18
- ◯ 19
- ◯ 20
- ◯ 21
- ◯ 22
- ◯ 23
- ◯ 24

How many hours is the shifts for your 4th year residents?

- ◯ 1
- ◯ 2
- ◯ 3
- ◯ 4
- ◯ 5
- ◯ 6
- ◯ 7
- ◯ 8
- ◯ 9
- ◯ 10
- ◯ 11
- ◯ 12
- ◯ 13
- ◯ 14
- ◯ 15
- ◯ 16
- ◯ 17
- ◯ 18
- ◯ 19
- ◯ 20
- ◯ 21
- ◯ 22
- ◯ 23
- ◯ 24

How many hours is the shifts for your 4+ year residents?

◯ 1
◯ 2
◯ 3
◯ 4
◯ 5
◯ 6
◯ 7
◯ 8
◯ 9
◯ 10
◯ 11
◯ 12
◯ 13
◯ 14
◯ 15
◯ 16
◯ 17
◯ 18
◯ 19
◯ 20
◯ 21
◯ 22
◯ 23
◯ 24

Does every clinical shift have a supervising emergency medicine specialist on site at all times?

◯ Yes
◯ No

Briefly describe how supervision is conducted ________________________________

Do you have didactic sessions for residents?

◯ Yes
◯ No

How many per month?(in hours) ________________________________

Do you have journal clubs?

◯ Yes
◯ No

How many per month? (in hours) ________________________________

Do residents lead journal club ?

◯ Yes
◯ No

Do residents give a lecture during didactics? I

◯ Yes
◯ No

In what year (s) of residency do they give a lecture? How often do they do it? ________________________________

In what year (s) of residency do they lead journal club? How often do they do it? ________________________________

Is the Dissertation research required part of EM residency

◯ Yes
◯ No

Is it required to be published?

◯ Yes
◯ No

How are your residents evaluated in the emergency department (Feedback, Exams, Other)?

____

How are your residents evaluated when in External rotation (Feedback, Exams, Other)?

____
